# Topics of Public Interest

**Published:** 1912-08

**Authors:** 


### TOPICS OF PUBLIC INTEREST

### Suicide Statistics

The following statistics are furnished by courtesy of the Bureau of the Census:

Registration area: calendar year 1910.

| Month | No. of Suicides. | Month | No. of Suicides. |
|---|---|---|---|
| Total | 8,590 | July | 777 |
| January | 641 | August | 714 |
| February | 581 | September | 706 |
| March | 856 | October | 710 |
| April | 713 | November | 684 |
| May | 757 | December | 648 |
| June | 803 | | |

Death rate per 100,000 population, 16.0.

The average number of suicides per month is about 716, the maximum, about 20% above the average occurring in March; the minimum about 20% below the average, in February. It is easy to see why the maximum should occur in March, when the weather is most trying and there has been an opportunity to feel the cumulative effects of financial strain, sickness and indoor work. The sudden drop in April may be accounted for by the removal of those contemplating suicide, by the actual performance of the deed in March. Allowing for this factor, there is a fairly regular decline, with no more fluctuations than might be expected from accidental reasons, throughout the year to February. One would naturally expect the minimum to occur during the summer when the weather, lessened financial burden, diminution of sickness and opportunities for recreation all tend to make life more tolerable. On the contrary, there is a decided rise for June and the summer months generally keep near the average. Possibly the slackening of business for the summer and consequent loss of work accounts for the June rise. It may be, too, that the greater opportunity to be by one's self in the country, and particularly beside the water, off-sets the favorable tendencies of the summer. The fall of the rate during the autumn and winter, is very likely due to revival of business.

Almost exactly 1% of all deaths occur from suicide. Excluding deaths in childhood, in which suicide is rare thought not so rare as formerly, and allowing for what are practically inevitable deaths by infection, etc., it is a safe estimate that one life in 20 or 25 is cut short by suicide. Everyone who contributes to making his fellow man's life more prosperous, happier and richer, is saving life. Everyone who is adding a cent a pound to the cost of sugar, meat, bread or any other commonly used food stuff, everyone who treats his fellow harshly and coldly, is taking life. Once in a great while, we note a case of suicide in which the individual is so useless—perhaps through no fault of his own—so depraved, so much in the way of others, that, humanly speaking, his death is a gain to humanity. In the great majority of instances, usefulness, happiness, self-respect, may be regained if the would-be suicide can be tided over a period of depression. And it is surprising what a small circumstance—a trival courtesy by a stranger, an interesting story, a kitten's antics, any little relief from introspection—can save a life in a critical period.

---

THE National Association for the Study and Prevention of Tuberculosis announces that nearly 4,000 additional hospital beds for consumptives in 29 states were provided during the year ending June 1st.

In the last five years, the hospital provision for consumptives has increased from 14,428 in 1907 to over 30,000 in 1912, or over 100 per cent. New York state leads in the number of beds, having 8,350 on June 1st; Massachusetts comes next with 2,800; and Pennsylvania, a close third with 2,700.

Only four states, Mississippi, Nevada, Utah and Wyoming have no beds whatever in special hospitals or wards for consumptives. Eight years ago, when the National Association was organized, there were 26 states in which no hospital or sanatorium provision for consumptives existed, and the entire number of beds in the United States was only 10,000.

It is stated, however, that there are from 250,000 to 300,000 indigent consumptives needing hospital care. Without wishing to underestimate the needs of this unfortunate class, and without having made a thorough study of the economics of the problem—if indeed there are reliable statistics available—we venture the opinion that the statement that there is accommodation for only one indigent consumptive in ten, is a trifle pessimistic. The tubercular mortality for the U. S. is commonly estimated at 150,000 a year and both the incidence and the mortality are gradually diminishing relatively to population so that some even claim a numeric decrease in spite of increasing population. It would seem that a hospital or sanitarium stay of two years is an ample allowance for each fatal case and that an equal allowance for non-fatal cases is generous. On this basis of calculation, there would be about 600,000 consumptives in the country, at any one time, requiring institutional care. It scarcely seems that half of them should be dependent on public charity; indeed, by age and sex incidence, more than half would normally be dependent on heads of families. However, we would not by any means discourage this worthy cause and there is not much use in disputing as to percentages when the need is so obviously far beyond the supply of institutional care.

---

### Typhoid Vaccination

The health department is to be commended for arranging for "free vaccination" against typhoid. In making Buffalo the first city in the United States to adopt such measures to combat the second most deadly of diseases, Dr. Fronczak is but doing his duty.

To announce Dr. Fonczak's plan as "free vaccination" is a misnomer. Buffalonians are paying and paying dearly for the innovation. The taxpayers are paying for the vaccine. But that is a small item in the expense that they are made to bear. They are paying for the conditions which make necessary vaccination against typhoid. They have been assessed millions of dollars to pay interest for the Emerald channel intake on the representation that it would make a pure water supply possible. Its failure is attested by the high mortality rate as shown in the statistics of the state department of health.

We have appropriated the above from the *Buffalo Express* of July 2, as additional evidence of the keen interest and cooperative spirit of the lay press in regard to medical matters. There is one point however, in which we are deceiving ourselves, and by deceiving others, injuring ourselves as a municipality. Granted that, so far as improving the water supply, the enormous expense of the new intake has been practically wasted; granted that our water is still liable to contamination; the fact remains that, relatively, Buffalo has very good water and, relatively, very little typhoid. We are personally convinced that at least 50% of our typhoid is due to infection outside the city limits.

### Changes at Syracuse University Medical School

This institution has added the following men to its corps of instruction: As assistant professor of bacteriology, Leverett Dale Bristol, A. B., M. D., Johns Hopkins, of St. Paul, Minn.; as instructor in the department of histology, Earl V. Sweet, A. B., M. D., Cornell, of Phœnix, N. Y.; as instructor in surgery, Albert G. Swift, M. D., Syracuse, of New York; as instructor in pathology, John W. Cox, M. D., of Syracuse. This position was secured by scholarship. At the suggestion of the dean, Colgate University has signified its intention of permitting students to take the senior year in a registered medical college *in absentia,* such students to receive the bachelor's degree upon the presentation of a certificate from the college of medicine attended, to the effect that his work has been done satisfactorily.

### Medical Study Tour

The Study Tour of the 9th Session of the *"Association Internationale de Perfectionnement Scientifique"* has been organized by the Central Board as follows:

*Concentration on the 8th August at Besancon.—Itinerary: Salzburg, Konigsee, Salt-mines of Berchtesgaden, Reichenhall, Tauern, Karawankes, Wochein, Adelsberg Grots, Agram, Danube, Passages of Kazan, Iron Doors, Bucarest, Constantinople (Pera-Stamboul-Scutari), Sofia, Belgrade, Fiume, Abbazia (the Nice of the Adriatic), Trieste, the Peninsula of Miramare, Venice. Separation on the 30th August at Aix-les-Bains.*

For particulars, write to "A. P. M. s." Office, 12 rue Francois-Millet, Paris XVIe.

---

We have received from Prof. M. A. Gilbert, of Paris, an announcement of special courses in clinical medicine and laboratory methods of diagnosis, beginning Monday, Sept. 23. The full announcement can be seen at this office. Paris has, in recent years, been neglected by American students; indeed, there has been a disposition to ignore French contributions to medicine. In perusing various medical works of the seventeenth and eighteenth centuries, the extent of accurate knowledge on the part of the French at that time, has been a source of wonder. Indeed, it has occurred to us that a revision of the erudite treatises on the lymphatic system, in the light of modern anatomy and physiology, is all that is necessary to develop a new field of clinical medicine. Post graduate study in London often proves unsatisfactory to the American, on account of the lack of punctuality and irregular hours kept. Probably a third of the population of the U. S. is German and this fact, as well as the phenomenal industry and skill of the Germans in developing the scientific aspects of medicine, account for the tendency of Americans to seek post graduate work in Germany or Vienna. Moreover, the German language has become familiar to many Americans not themselves of German origin. The French in America have, too often, forgotten their ancestry and failed to learn their mother—or perhaps we should say their several-great grandmother—tongue. From personal experience, we can recommend the Parisian clinics, largely on account of the courtesy shown American students, though we would not imply any lack of such courtesy elsewhere, but also because of the quite American spirit of enterprise and energy, of practicality, and of keeping the main issue of the patient's welfare uppermost.

---

The New York City Society for the Prevention of Cruelty to Children has found, that of about 700,000 children of school age in the present year, over 1% are feeble minded. It recommends a preliminary investigation of the mental condition of all school children, the establishment of an institution for their care, and more efficient means of excluding defective immigrants. The last recommendation, though theoretically wise, impresses us as very difficult of execution. Imagine yourself trying to investigate the mentality of a shy and probably frightenend child, speaking the rudiments of a foreign language, probably a dialect of that, necessarily ignorant of existing conditions, brought up in a very simple community where peculiar standards of social life prevail. How could you determine in a necessarily brief interview and examination whether the child was or was not feeble-minded—barring, of course, conspicuous defects? A point of the utmost importance, often overlooked, is that it is not the out-and-out idiot, with receding forehead, distorted features and obvious lack of judgment that needs protection, so much as the high-grade imbecile who, if a boy, is easily led into crime of which he has no proper realization and who, if a girl, is led astray sexually when the obvious idiot would be protected by her very repulsiveness or her plain appeal for chivalrous protection.

That sounds may by communicating vibrations to find particles, as of sand, or to a flame, or to an impressionable material as in the phonograph, yield visual impressions, has long been known. Dr. H. B. Williams of Columbia, has recently been carrying on a series of experiments with reference to the photographing of the vibrations of heart sounds, employing a string galvanometer, in connection with a microphone, and the movements of the quartz of the former are magnified by being projected on a screen, the shadow on which is photographed.

---

Itinerants, calling themselves doctors and druggists, are at large in Buffalo, selling drugs and even diagnosing disease and prescribing. The Health Department has issued a public warning.

---

Buffalo is having an epidemic of measles, amounting to more than a thousand cases. Otherwise, there is very little sickness.

---

Tonawanda finds that considerable sewage has been directed into the State Ditch and the ditch has been obstructed by drift wood and other debric. The obstructions have been cleared away but there is a legal question as to who is responsible for the drainage of sewers, property owners claiming that they have been accepted by the city at previous inspections and that any expense of changes must be borne by the city.

---

Niagara County Supervisors have voted $40,000 for a tuberculosis hospital. The annual mortality from tuberculosis in the county is about 90. Drs. Leo, Wolf and Scott of Niagara Falls, and Dr. C. N. Palmer of Lockport spoke in favor of the hospital. Mr. C. F. Nelbach of the Charities Aid Association stated that 24 of the 61 counties of the state have either established or voted to establish county hospitals.

---

The Public Service Commission has arranged for a simplified and more reasonable system of express rates. The extortion of our express companies and their influence in preventing the extension of a parcels post, has long been a national scandal. Recounty is about 90. Drs. Leo-Wolf and Scott of Niagara Falls, 80 miles away. It cost more than to send them singly at full postal rates, to any distance in the U. S., Canada and Mexico, Personally, we think it would have been better to have ignored the express charges altogether and to have provided for a gradual but rapid increase in weight and size of fourth-class mail matter, at reduced pound rates. An immediate change would have swamped the postal service.

---

A Medical Shoplifter.—The daily press has recently told a sad story of a well educated and formerly successful woman physician, of Boston who, after 25 years' practice has been fined and placed under the supervision of a probation officer for shoplifting. Such an occurrence naturally throws discredit on the entire profession but there are two mitigating circumstances: she stole honestly and made herself amenable to the law instead of laughing at it as a "borrower"; and, when arraigned, she pleaded guilty and scorned the subterfuge of kleptomania.

---

How the Profession Changes.—A few nights ago, while waiting at a hospital for an operator, the editor in his alternate role, amused himself in studying one of those group pictures of prominent members of the profession, by which certain firms enrich themselves occasionally. This group included 158 Buffalo physicians in 1899. 27 were dead, 10 had departed for less remote places, 1 is retired, 1 has gone into another business. Almost exactly a quarter removed in 12 1-2 years.

The list of physicians for Buffalo, ten months ago (Sept. 15, 1911, prepared with the utmost care up to that date) numbered 661. This was a list of those entitled to practice, included some internes, several married women physicians, and quite a good many others not actually in practice. At a rough estimate, those really in private practice last fall numbered about 600. At least nine were engaged in other businesses and seven had retired. Since then, 15 have moved away, and four have died. Our subscription records show five new arrivals in the city but we believe several others have also located here.

---

### Surgical Aspects of the D. L. & W. Wreck at Corning

The Buffalo Medical Journal took immediate steps to secure a full surgical account of the disaster but was unable to obtain more than a few details. The killed are said to have sustained fractures of the skull, in all instances. The seriously injured were, for the most part, cases of head injury, especially fractures of the jaw. One man had a multiple fracture of the upper jaw, a fracture and flattening of the nose and bled profusely from the mouth. There was also a severe facial laceration, crossing one eye but not involving the eye itself; and several fractures of the left leg between the hip and ankle. He survived the shock and the prognosis is good. Three other cases of head injury involved loss of consciousness. One, a woman remained unconscious for several days and a boy is still irrational at times but all three are regarded as progressing favorably. At Elmira, several fracture cases are being cared for. One case, that of a woman, included spinal paralysis and a fractured clavicle but is recovering.

The Lockport City Hospital is liable to be closed on account of lack of funds, there being apparently no legal authority for an additional appropriation.

---

The Corning typhoid epidemic has abated. The plan to bring water from Pennsylvania, as previously announced, has been abandoned in favor of deep local wells. An abundant supply of soft water has been struck by test wells, at a depth of 100 feet, on the Fuller farm.

---

The Tonawanda *Evening News* opposes the plan of the State Conservation Commission to build a storage reservoir on Tonawanda Creek, though admitting its usefulness for Batavia, Lockport, Albion, Medina, etc. This reservoir is estimated to afford a supply for 100,000 population. The *News* points out that the combined population of the Tonawandas and Niagara Falls is already nearly 60,000 and that it will soon exceed the capacity of any such storage scheme while the inforcement of the Federal regulations as to the pollution of the Niagara River is inevitable and will afford an inexhaustible supply of relatively pure water for the entire Frontier.

---

The N. Y. State Dept. of Health has organized a state-wide campaign of sex education among women and girls, to be carried on largely by women physicians.

---

A LEPER IN BUFFALO.—Sam Isen, a Russian Jew, who came to this country three years ago, was confined by the Bay City, Mich., authorities last spring as a leprosy suspect. Two weeks ago he escaped with the intention of returning to Russia (which scarcely corroborates the prevalent opinion regarding persecution in the latter country) and has been lodging in a public shelter in Buffalo for some time, waiting for funds. Dr. L. F. Anderson located him, with the help of a policeman and took him to the Ernest Wende Hospital where he is detained. Dr. W. S. Goodale reports that, to the casual observer, the case would not be obvious as the face shows no deep lesions. Macules and tubercles are, however, visible on the trunk. Bacilli were identified.

